# Mikkeli Osteoporosis Index Identifies Fracture Risk Factors and Osteoporosis and Intervention Thresholds Parallel with FRAX

**DOI:** 10.4061/2011/732560

**Published:** 2011-05-04

**Authors:** Ville Juhana Waris, Joonas P. Sirola, Vesa V. Kiviniemi, Marjo T. Tuppurainen, V. Pekka Waris

**Affiliations:** ^1^Department of Orthopaedics, Mikkeli Central Hospital, 50100 Mikkeli, Finland; ^2^Department of Orthopaedics, Kuopio University Hospital, 70211 Kuopio, Finland; ^3^Bone and Cartilage Research Unit (BCRU), Clinical Research Center, Kuopio University, 70211 Kuopio, Finland; ^4^Department of Statistics, Kuopio University, 70211 Kuopio, Finland; ^5^Department of Obstetrics and Gynaecology, Kuopio University Hospital, 70211 Kuopio, Finland

## Abstract

Osteoporosis Index (MOI) was developed from Fracture Index (FI), a validated fracture risk score, to identify also osteoporosis. MOI risk factors are age, weight, previous fracture, family history of hip fracture or spinal osteoporosis, smoking, shortening of the stature, and use of arms to rise from a chair. The association of these risk factors with BMD was examined in development cohorts of 300 Finnish postmenopausal women with a fracture and in a population control of 434 women aged 65–72. Validation cohorts included 200 fracture patients and a population control of 943 women aged 58–69. MOI identified femoral neck osteoporosis in these cohorts as well as the Osteoporosis Self-Assessment Tool (OST). In the pooled fracture cohort, the association of BMI-based FRAX fracture risk with MOI was good. After BMD measurement, MOI identified well FRAX hip fracture risk-based Intervention Thresholds (ITs) (AUC 0.74–0.90).

## 1. Introduction

Osteoporosis prediction rules attempt to select patients for bone densitometry. A recent review updates the performance of externally validated instruments that reported performance characteristics in Cochrane Database between 2001 and 2009 [1]. Twenty-three studies of 14 instruments to predict low BMD reported AUC estimates ranging mostly between 0.6 and 0.8. Of these, Osteoporosis Self-Assessment Screening Tool (OST) includes only age and weight but has similar area under the ROC-curve (AUC) estimates as the other more complicated instruments. Its validity in identifying osteoporosis has been confirmed in multiple independent population cohorts both in men and women [1–3]. 

Most fractures occur in patients with normal or osteopenic bone mass and instruments that predict low bone density correlate only modestly with clinical fractures [1]. Fracture risk assessment tools use clinical risk factors (CRF) to predict fractures, and combining bone densitometry with risk score usually results in higher AUC estimates [1, 4]. Again, instruments with fewer risk factors often do as well as those with more [1]. 

Recent meta-analyses and reviews have revealed the main BMD-independent CRFs for osteoporosis fractures: increasing age, low weight, previous fracture, family history of osteoporosis fracture, smoking, glucocorticoid therapy, neuromuscular disorders, and alcohol excess [5–10]. 

Fracture Index (FI) is a validated risk score for fracture prediction in white women over the age of 65. It includes six CRFs: increasing age over 65, fracture after age 50, maternal hip fracture, weight below 58 kg, smoking, and the use of arms to rise from a chair test [11]. The recommendations of the National Osteoporosis Foundation (NOF) for risk assessment contain the first 5 of these factors [12]. Also the recent multiethnic Women's Health Initiative (WHI) algorithm predicted hip fracture within 5 years as well as BMD. The WHI CRFs include the 5 factors above and, additionally, general health, race, physical activity, corticosteroid use, and diabetes [13]. 

WHO fracture risk assessment tool FRAX integrates BMD with CRFs: age, weight/height (BMI), previous fracture, parent fractured hip, current smoking, use of glucocorticoids, use of alcohol 3 or more units/day, rheumatoid arthritis, and causes of secondary osteoporosis [14]. 

The aim of the present study was to develop from FI a risk score which identifies both fracture risk factors and low BMD in Finnish population. We named this simple additive score Mikkeli Osteoporosis Index (MOI), and compared the correlation of MOI, FI, and OST with BMD. We further compare the above scores with FRAX fracture risk and the concordance of MOI with FRAX to identify Intervention Thresholds (ITs) proposed by the WHO Collaborating Group [15–17]. 

## 2. Materials and Methods

To obtain both epidemiological and clinical validity, we used two independent development cohorts (Mikkeli Central Hospital fracture patient cohort and Kuopio Fracture Prevention Study (FPS) population cohort) and two validation cohorts (another Mikkeli fracture patient cohort and Kuopio population-based Osteoporosis Risk factor and Prevention study (OSTPRE) cohort). Fracture patients and population cohorts were recruited by separate research teams during separate time periods. 

### 2.1. Mikkeli Central Hospital Patient Cohorts (Development and Validation Cohorts 1)

Between 1.1.2002 and 30.4.2005 a total of 698 consecutive female low energy fracture patients, aged 45–79, who had fallen on the same level or from a height of less than one meter were treated in Mikkeli Central Hospital, Finland. Development Cohort 1 included 300 of these, who accepted to participate in the study. Patients with dementia, psychic instability, known secondary osteoporosis (type I diabetes, rheumatoid arthritis, long-term glucocorticoid use, malabsorptive syndromes) and women taking bone active medications other than ovarian hormones, calcium, or vitamin D were excluded. Thus, 180 out of 200 radius fracture patients (90%) were prospectively included in the study, as well as 24 patients with fracture of the proximal humerus (40%), 21 clinical spine (87%), 23 hip (7%), and 52 other extremity fracture patients (61%). Patients with fractures of hand or foot were excluded from the study. 

The patients filled in a questionnaire with the FI risk factors, including family history of hip fracture or senile spinal hump with shortening, and recalled height at age 30. Their weight was measured with a digital calibrated scale and the height with a calibrated wall meter. The ability to rise from a chair without use of arms was tested. Hospital staff nurses registered and two osteoporosis nurses recorded the data. BMD of lumbar spine and proximal femur was measured with Lunar DPX-IQ. Patients provided an informed, written consent to participate in the study. The study was approved by the local ethics committee. 

The next 200 consecutive low energy fracture patients, treated in Mikkeli Central Hospital between 1.5.2005 and 1.10.2007, were used as a clinical validation cohort (Validation Cohort 1) for MOI. These included 104 radius fracture patients, 20 with fracture of the proximal humerus, 9 clinical spine, 5 hip, and 62 other low energy extremity fracture patients. The process of recruitment and data collection was otherwise identical with that of the first 300 patients of the Development Cohort 1. 

### 2.2. Population-Based Development and Validation Cohorts 2

These study populations included two separate independent random densitometry samples selected from the prospective Osteoporosis Risk Factor and Prevention (OSTPRE)-study cohort: Development Cohort 2 and Validation Cohort 2. 

The OSTPRE cohort was established in 1989 by selecting all women born in 1932–1941 and resident in Kuopio Province, Finland (*n* = 14220) [18]. 

The baseline postal questionnaire of the OSTPRE cohort included questions about health disorders, medication, use of hormone therapy (HT), gynaecological history, nutritional habits, calcium intake, physical activity, alcohol consumption, smoking habits, and anthropometric information [18]. Five-year (in 1994–97), ten-year (1999–2001), and fifteen-year (2000–2003) follow-up questionnaires were sent to the 13100 women who responded to the questionnaire at baseline, with responses of 11954 (5-year), 11537 (10-year), and 10926 (15-year). 

A subsample (*n* = 3222) of the 13100 baseline respondents was resourced for central bone density measurements. Of these, the randomised population-based sample consisted of 2025 women. In all, 1873 women of the random part underwent the 5-year and 10-year bone density measurements with Lunar DPX-IQ. Serial valid measurements for neck of femur and lumbar spine were recorded for 1438 women in both baseline and follow-up measurements. 

Patients with metallic implants or severe bone deformities, including osteoarthritis with significant osteophytes, were excluded after a systematic manual review of densitometry reprints by the research team physicians. Hysterectomized women, for whom it was not possible to define menopausal status, and premenopausally bilaterally ovariectomized women were additionally excluded. Accordingly, the final Validation Cohort 2 consisted of the OSTPRE cohort women with complete results of the BMD measurements and FI risk factors in the 10-year follow-up study (*N* = 943, age 58–69 years). The FPS population (*n* = 5407) was randomly selected in 2003 from the OSTPRE baseline respondents (*n* = 13100) with a purpose to determine the effect of vitamin D and calcium in fall and fracture prevention in postmenopausal women. The inclusion criteria for FPS study were age over 65 years, living in Kuopio province at the inclusion time, and not belonging to the original OSTPRE BMD-measurement sample. 3432 women of the 5407 (63.5%), willing to participate in the prospective vitamin D and calcium trial, were randomized into two groups of equal size. FPS Cohort, a subsample of 434 women, aged 65–72 years, was randomly selected at the baseline (Development Cohort 2) and underwent detailed measurement program. Risk factor analysis included standardized height and weight measurements, balance tests, grip strength, and food, smoking, and physical activity diaries. The adult height at age 30 and all recalled fractures during adulthood were registered. A half way squatting test was used instead of the use of arms to rise from a chair test. The baseline values of BMD and risk factors have been described previously [19]. 

In FPS and OSTPRE studies, two specially trained nurses carried out DXA measurements in Kuopio University Hospital. Quality standards were tested on daily basis. The short-term reproducibility of this method has been shown to be 0.9% for lumbar spine and 1.5% for femoral neck BMD measurements. The long-term reproducibility (CV) of the DXA instrument, as determined by regular phantom measurements, was 0.4% [20]. BMD results were expressed as T-scores based on the manufacturer's reference database. 

## 3. Statistical Methods 

The characteristics of the development cohort—BMD, age, weight, height loss, and FI risk factors—were compared using t-test and chi-square test. To compare potential risk factors by age, the fracture cohort was dichotomized into age groups of 45–64 and 65–79 years. 

BMD, age, weight, and height loss were examined as continuous variables in the development cohorts. Other CRFs were dichotomized (yes/no) and were examined with appropriate univariate statistical analyses. The age was categorized into 5-year thresholds like in FI. Weight was categorized into 5 groups to examine linearity of the association of weight and BMD. Height loss was categorized into 3 groups based on contextually and statistically meaningful association with BMD. Continuous and categorized variables were compared with linear regression and ANOVA. To keep the ratio of the BMD-independent fracture risk factors stable in the final model, we multiplied the original FI risk factors by 2 and aligned the age thresholds with those of FI in the age range of 70–79. We named this simple additive score Mikkeli Osteoporosis Index (MOI) and compared the correlation of MOI, FI, and OST with BMD. We plotted the ROC curves of MOI, FI, and OST for identifying osteopenia (T-score ≤ −1.5 or ≤ −2.0) and osteoporosis (T-score ≤ −2.5 either in femoral neck, total hip, or spine (L 2–4 area)) both in the development and validation cohorts. The difference between the AUC values was tested with univariate z-score test. The results were considered significant at *P* < .05 level. We used Excel 96 and SPSS Windows 11.5 statistical programs. 

To obtain true clinical relevance, we pooled the fracture cohorts and calculated with FRAX-UK-tool the Body Mass Index-based FRAX 10-year major osteoporosis fracture risk (FRAX-BMI) of each fracture patient (*N* = 500). Using regression analysis, we compared MOI, FI, and OST with FRAX-BMI. 

We further compared the concordance of MOI, after BMD-measurement, to identify BMD-based FRAX 10-year fracture risk (FRAX-BMD) ITs in the pooled fracture cohort. We used the smoothed 10-year hip fracture probabilities presented for the UK and Australia by Borgström et al. [17], which were approximated to integer. For MOI, we used three groups: low risk (no treatment), intermediate risk (treatment based on BMD result), and high risk (treatment without BMD measurement) (Table 4). We calculated the identification characteristics between the three MOI risk groups and the seven risk thresholds of FRAX-BMD. 

## 4. Results and Discussion

### 4.1. Development of the Score

The mean age of the Mikkeli Central Hospital 300 fracture patients (Development Cohort 1) was 65 ± 9 years. Femoral neck BMD (BMD-N) was normal (T-score over −1 SD) in 11–18% of the patients and osteoporotic (T-score ≤ −2.5 SD) in 35–40%. Fracture patients under the age of 65 had more osteoporosis in their family and smoked more than patients above age 65 (Table 1). 

Univariate analysis was performed both in fracture patient cohort (Development Cohort 1) and in the FPS population (Development Cohort 2). Femoral neck and total hip BMD were associated with weight, age, height loss, and previous fracture, in decreasing order of importance (Table 2). The association of BMD with age was linear, whereas the association of BMD with weight disappeared nonlinearly above 80 kg. Categorizing continuous variables (age, weight, and shortening) did not significantly change the association. Hip BMD was associated, additionally, with the use of arms to rise from a chair test and family history, but with smoking only in fracture patients above age 65 (Table 2). 

In multivariate linear regression models, these associations with BMD remained stable; only shortening of the stature lost its value in fracture patient cohort (data not shown). 

Based on the above analyses, we included 7 factors in MOI: *age *55–59/60–64/65–69/70–74/75 years. (1/2/3/4/6 risk points), *weight *below 80/71/64/58 kg (1/2/3/4 p), *previous adult fracture*, *family history of hip fracture or spinal osteoporosis *and *smoking *(2 p each), *shortening *by 3/5 cm (1/2 p), and *use of arms to rise from a chair *(2 p), max 20 p. 

The AUC values to identify osteoporosis in the femoral neck in the different cohorts are presented in Table 3. In the total hip and spine (L 2–4) areas, the AUCs for osteoporosis in the different cohorts were 0.72–0.78 and 0.66–0.74 with MOI, 0.59–0.71 and 0.59–0.65 with FI and 0.56–0.75 and 0.59–0.73 with OST, correspondingly. The differences between scores were mostly not statistically significant. 

In the OSTPRE validation controls at age 58–69, the AUC increased significantly from osteopenia to osteoporosis (AUC BMD-N −1.5/−2.5: MOI 0.63/0.79, FI 0.59/0.76 and OST 0.59/0.74). In the fracture validation patients, the scores operated identically at BMD-levels −2.5 and −2, but not at all at BMD-level −1.5 (data not shown). 

### 4.2. Comparison of MOI with FRAX in the Pooled Fracture Cohort

**Figure 1:**
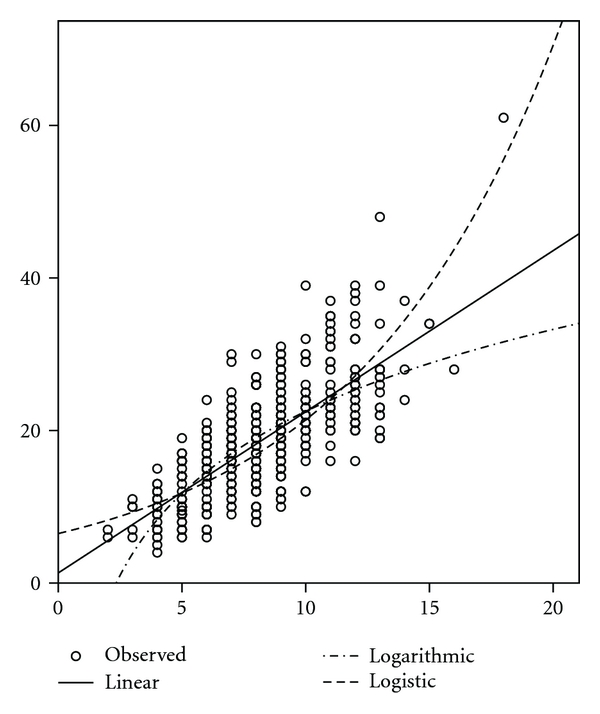
Correlation of MOI with Body Mass Index-based FRAX 10-year major osteoporosis fracture risk in the pooled fracture cohort (*N* = 500) (*R* ^2^ = 0.54,*F* = 596). (*x* = MOI, *y* = FRAX).

In regression analysis, the association of MOI with FRAX-BMI was highly significant in the pooled fracture cohort (*R* ^2^ = 0.54, *F* = 596,*N* = 500, Figure 1). The association of FI and OST with FRAX-BMI was poor (*R* ^2^ for FI versus OST in the pooled fracture cohort were 0.08 versus 0.01). 

The smoothed intervention thresholds by FRAX-BMD and the corresponding MOI thresholds are presented in Table 4. The characteristics of diagnostic concordance between MOI and FRAX-BMD in the pooled fracture cohort are presented in Table 5. The sensitivity of MOI was high and the specificity moderate. Those patients in the fracture cohort which were identified as false positives by MOI fulfilled the FRAX treatment threshold with a mean of 77 (55–93)%. 69% (16 out of 26) of the false negative patients in the fracture cohort, unidentified by MOI, had BMD > −1.5. 

Our aim was to validate a score that identifies both low BMD and independent fracture risk factors. The score was developed within low energy fracture patients in Mikkeli, Finland, with the assistance of a separate population-based control group (FPS). It was validated in two independent cohorts, both in fracture patients and in population-based controls (OSTPRE). The risk score, named MOI, is a modification of the previously introduced FI. MOI identified both low BMD and classifies patient ITs in concordance with FRAX. 

There were limitations in the development of MOI. The participation rate of hip or humerus fracture patients in this prospective study was low because of high age, frailty, or dementia. The development and validation cohorts were independent of each other but were of the same geographical region. The population-based FPS development cohort had only a narrow age range, and therefore the effect of age on BMD could be analysed only in fracture patients. The size of the fracture Validation Cohort 2 was limited, but it represents typical clinical white female patients in which osteoporosis CDRs would be applied. Both control groups were representative population-based cohorts with a high participation rate and long followup. Two specially trained nurses registered and collected the control group data, whereas staff nurses registered and two osteoporosis nurses only collected the corresponding data in the clinical fracture series. Also, misinterpretations of the densitometry reprints were excluded in the population cohorts, which may explain the higher AUCs for osteoporosis identification in the OSTPRE population controls. 

FI registers only low weight < 58 kg. In our study, the relation between weight and BMD was nonlinear. In WHO meta-analysis, low BMI below 20 had a twofold hip fracture risk compared to BMI of 25. The risk levelled off in nonlinear fashion as BMI increased to 30 [8]. FI includes postmenopausal fractures above age 50, and NOF treatment recommendations include fractures above age 40 [11, 12]. FRAX and MOI include all previous adult fractures because these indicate an increased risk for later fractures (RR 1.8–2.0 both in men and women) [6]. 

FI and FRAX include only maternal/parental hip fractures, while MOI includes hip fracture and spinal osteoporosis in all first-degree relatives. In WHO meta-analysis, any parental or sibling osteoporosis fracture increased the risk of subsequent hip and osteoporosis fractures independent of BMD (RR 1.5–2.3) [7]. Also, our research team recently identified an association between increased fracture risk in perimenopausal women and their sisters' fracture history [21]. 

Awareness of height loss and changing body profile is considered to increase patient compliance [22]. Height loss by 2–5 cm increases the risk of hip fractures and risk of silent and clinical vertebral fractures [23, 24]. Height loss was independently predictive of hip fractures in the WHI study [13] and is a risk factor in the EPOS fracture algorithm [25]. 

MOI, FI, and NOF treatment recommendations all have the same five CRFs, which have been identified also in recent meta-analyses and reviews [6–10]. They were also good predictors of actual occurrence of fractures in a cohort of postmenopausal women followed prospectively for up to 22 years [26]. The FI risk factors identified fractures also in the prospective multisite Canadian CANDOO clinical patient cohort [27]. 

The FRAX Tool is based on the above meta-analyses and has been validated in large independent prospective cohorts. It calculates the 10-year probability of fractures in several countries [15, 17]. ITs based on major osteoporosis fracture risk instead of hip fracture risk seem to increase sensitivity of identification in younger age groups [16]. 

However, recent data from prospective FIT and SOF-study population cohorts of elderly white women suggest that even more simple models, based on age, femoral neck BMD, and fracture history predict clinical fracture as well as more complex FRAX models [28, 29]. These findings, however, require confirmation in other cohorts of younger women or men and different geographic settings. Our prospective fracture cohorts represent typical clinical white female patients in which osteoporosis CDRs would be applied. 284 patients in the pooled cohort (*N* = 500) had a radius fracture. In these patients, the three risk groups of MOI seem, after BMD measurement, to identify ITs similarly to the seven age/risk thresholds of FRAX-BMD. The majority of false negative patients in the fracture cohort, unidentified by MOI, had BMD > −1.5. Bisphosphonate therapy has not been demonstrated to be effective with femoral neck T-scores better than −1.5. In women with osteopenia below T − 1.5 therapy is cost-effective in USA after additional BMD-independent fracture risk factors that confer a BMD-adjusted relative fracture risk of 2.0 or higher [30]. 

MOI is currently in clinical use in the majority of Finnish central hospital districts. Its advantage is that it identifies osteoporosis and fracture risk factors with a single figure: low risk, MOI 0–5/6 (no treatment), intermediate risk (treatment based on BMD result), and high risk, MOI > 11/12 (treatment without BMD measurement). The intermediate risk group to identify patients for BMD measurement can be adjusted according to national variations in fracture incidence and diagnostic and treatment resources. 

## 5. Conclusions

MOI identifies osteoporosis and fracture risk factors with a single figure and, after BMD measurement, Intervention Thresholds in concordance with FRAX.

## Figures and Tables

**Table 1:** BMD and prevalence of risk factors (%) in the development patient cohorts.

	Development Cohort 1 (Fracture patients)		Development Cohort 2 (FPS)
	Age < 65 y, *N* = 141	Age 65 y, *N* = 159	Age 65–72 y*N* = 434
Age (mean, SD)	57 5 y	71 4 y	68 2 y
BMD-N	−0.9, 0.3***	−1.6, 0.9	−1.6, 0.9
BMD-H	−1.2, 0.4***	−1.5, 0.9	−1.5, 0.9
BMD-S	−1.4, 0.9	−1.5, 1.3	−1.5, 1.3

	%	%	%
Osteoporosis	35***	40***	14
Osteopenia	47	49	47
BMD normal	18***	11***	39
Weight ≤ 57 kg	18***	13	8
Previous fracture		15	
Family history of hip fracture or spinal osteoporosis	34***	15	14
Current smoker	18***	4	5
No regular exercise/walking	18*	16**	10
Shortening 5 cm	2	22***	4
Shortening 3-4 cm	14	26*	18
Use of arms to rise from a chair	11	18***	6

*P < .05, **P < .01, ***P < .001, significant difference against FPS cohort.

BMD-N: bone mineral density (T-score), femoral neck.

BMD-H: bone mineral density (T-score), proximal femur.

BMD-S: bone mineral density (T-score), spine L 2–4.

FPS: Fracture Prevention Study.

**Table 2:** Proportion of variance in BMD in Development Cohort 1 (fracture patients, *N* = 300, age 45–79) and Development Cohort 2 (FPS, *N* = 434, age 65–72). *R* ^2^ value (%), explained by the risk factors in univariate regression models, and by MOI, FI, and OST-scores.

Fracture patients			
*N* = 300	*R* ^2^; BMD-N	*R* ^2^; BMD-H	*R* ^2^; BMD-S
Weight, continuous	0.14***	0.19***	0.12***
Weight, categorized	0.13***	0,17***	0.12***
Age, continuous	0.13***	0.07***	0.01
Age, categorized	0.12***	0.07***	0.0
Shortening, continuous	0.08***	0.06***	0.02*
Shortening, categorized	0.05***	0.03*	0.01
Family history	0.01	0.0	0.0
Smoking	0.0	0.01	0.01
Rise from a chair test	0.0	0.02*	0.0
MOI	0.22***	0.22***	0.0
FI	0.08***	0.07***	0.0
OST	0.25***	0.25***	0.0

FPS			
*N* = 434	*R* ^2^; BMD-N	*R* ^2^; BMD-H	*R* ^2^; BMD-S

Weight, continuous	0.05***	0.12***	0.09***
Weight, categorized	0.06***	0.12***	0.09***
Age, continuous			
Age, categorized			
Shortening, continuous	0.01*	0.01	0.01
Shortening, categorized	0.03***	0.03***	0.02**
Fracture history	0.03***	0.03***	0.04***
Family history	0.0	0.01*	0.0
Smoking	0.0	0.0	0.01
Rise from a chair test	0.0	0.02*	0.0
MOI	0.11***	0.17***	0.11***
FI	0.04***	0.06***	0.03**
OST	0.06***	0.12***	0.19***

MOI: Mikkeli Osteoporosis Index; FI: Fracture Index; OST: Osteoporosis Self-Assessment Tool. Other abbreviations, see Table 1.

**Table 3:** AUC values (standard error) for MOI, FI, and OST with osteoporosis (BMD-T-score ≤ −2.5) at the femoral neck in the development FPS (age 65–72, *n* = 434) and fracture cohorts (age 45–79, *N* = 300) and in the validation fracture cohort (*N* = 200) and OSTPRE validation cohort (age 58–69, *n* = 943).

	FPS cohort age 65–72	Fracture development cohort	Fracture validation cohort	OSTPRE cohortage 58–69
MOI	0.67 (0.06)	0.75 (0.03)	0.67 (0.07)	0.79 (0.04)
FI	0.56 (0.06)	0.68 (0.04)	0.53 (0.08)	0.76 (0.04)
OST	0.63 (0.06)	0.79 (0.03)*	0.62 (0.07)	0.74 (0.05)

*P < .05, significant difference between OST and FI.

MOI: Mikkeli Osteoporosis Index.

FI: Fracture Index.

OST: Osteporosis Self-Assessment Tool.

**Table 4:** Intervention thresholds by FRAX-BMD presented for UK and Australia (smoothed 10-year hip fracture probabilities, %) and the corresponding MOI thresholds, used for diagnostic comparison.

	FRAX risk (%)	
Age, y.	UK	Australia
50	1	2
55	2	3
60	3	4
65	4	5
70	5	7
75	6	10
80	7	10

MOI score		

No treatment	0–4	0–5
Treatment by BMD	5–11	6–12
Fracture patients	T < −1.5	T < −2
Patients without fracture	T < − 2	T < −2.5
Treatment without BMD	12	13

**Table 5:** Characteristics of diagnostic concordance between MOI and FRAX-BMD to identify Intervention Thresholds presented for UK and Australia (see Table 4).

	Pooled fracture patients (*N* = 500)	
MOI characteristics	UK	Australia
False−	26	16
True−	205	327
True+	165	96
False+	7	61
LR+	2.7	5.4
LR−	0.14	0.17
Sensitivity (%)	91	86
Specificity (%)	66	84
AUC (%)	74	90

LR+: positive likelihood ratio.

LR−: negative likelihood ratio.

AUC: area under the ROC curve.
