# Corrigendum to “Mikkeli Osteoporosis Index Identifies Fracture Risk Factors and Osteoporosis and Intervention Thresholds Parallel with FRAX”

**DOI:** 10.1155/2017/3074151

**Published:** 2017-08-20

**Authors:** Ville Juhana Waris, Joonas P. Sirola, Vesa V. Kiviniemi, Marjo T. Tuppurainen, V. Pekka Waris

**Affiliations:** ^1^Department of Orthopaedics, Mikkeli Central Hospital, 50100 Mikkeli, Finland; ^2^Department of Orthopaedics, Kuopio University Hospital, 70211 Kuopio, Finland; ^3^Bone and Cartilage Research Unit (BCRU), Clinical Research Center, Kuopio University, 70211 Kuopio, Finland; ^4^Department of Statistics, Kuopio University, 70211 Kuopio, Finland; ^5^Department of Obstetrics and Gynaecology, Kuopio University Hospital, 70211 Kuopio, Finland

In the article titled “Mikkeli Osteoporosis Index Identifies Fracture Risk Factors and Osteoporosis and Intervention Thresholds Parallel with FRAX” [1], there was an error regarding the FRAX® tool, which should be clarified as follows.

The article notes: “WHO fracture risk assessment tool FRAX integrates BMD with CRFs: age, weight/height (BMI), previous fracture, parent fractured hip, current smoking, use of glucocorticoids, use of alcohol 3 or more units/day, rheumatoid arthritis, and causes of secondary osteoporosis [14].” However, the World Health Organization (WHO) did not develop, test, or endorse the FRAX tool or its recommendations [2]. The metabolic bone disease unit at the University of Sheffield that developed FRAX was a WHO Collaborating Centre from 1991 to 2010, but treatment guidelines must undergo a formal process before they can be endorsed by the WHO.
